# Mechanisms Ensuring Endothelial Junction Integrity Beyond VE-Cadherin

**DOI:** 10.3389/fphys.2020.00519

**Published:** 2020-05-21

**Authors:** Cao Nguyen Duong, Dietmar Vestweber

**Affiliations:** Department of Vascular Cell Biology, Max Planck Institute for Molecular Biomedicine, Münster, Germany

**Keywords:** VE-cadherin, adhesion molecules, endothelial junctions, vascular permeability, angiogenesis

## Abstract

Endothelial junctions provide blood and lymph vessel integrity and are essential for the formation of a vascular system. They control the extravasation of solutes, leukocytes and metastatic cells from blood vessels and the uptake of fluid and leukocytes into the lymphatic vascular system. A multitude of adhesion molecules mediate and control the integrity and permeability of endothelial junctions. VE-cadherin is arguably the most important adhesion molecule for the formation of vascular structures, and the stability of their junctions. Interestingly, despite this prominence, its elimination from junctions in the adult organism has different consequences in the vasculature of different organs, both for blood and lymph vessels. In addition, even in tissues where the lack of VE-cadherin leads to strong plasma leaks from venules, the physical integrity of endothelial junctions is preserved. Obviously, other adhesion molecules can compensate for a loss of VE-cadherin and this review will discuss which other adhesive mechanisms contribute to the stability and regulation of endothelial junctions and cooperate with VE-cadherin in intact vessels. In addition to adhesion molecules, endothelial receptors will be discussed, which stimulate signaling processes that provide junction stability by modulating the actomyosin system, which reinforces tension of circumferential actin and dampens pulling forces of radial stress fibers. Finally, we will highlight most recent reports about the formation and control of the specialized button-like junctions of initial lymphatics, which represent the entry sites for fluid and cells into the lymphatic vascular system.

## Introduction

Intercellular junctions enable endothelial cells to form multicellular structures that develop into sprouts and primitive vascular tubes. Through remodeling into arteries, capillaries and veins, complex vascular structures evolve associated with perivascular cells (Adams and Alitalo, 2007). Endothelial cells then form the inner lining of a complex and diverse vasculature, where endothelial junctions guard and control vascular permeability and leukocyte trafficking (Vestweber et al., 2014). A multitude of cell adhesion molecules supports and controls endothelial junction integrity in the adult organism, thereby guarding traffic of molecules and cells across the vessel wall (Trani and Dejana, 2015).

These adhesion molecules form tight junctions and adherens junctions which are less clearly separated in endothelial cells than it is known for epithelia. The vasculature in different organs varies in many aspects due to organ specialization (Aird, 2007a,b; Augustin and Koh, 2017; Potente and Mäkinen, 2017). In accordance to this, the molecular requirements for endothelial junction integrity diverges between tissues. It emerged recently that this is also the case for lymphatic vessels in different organs.

**FIGURE 1:**
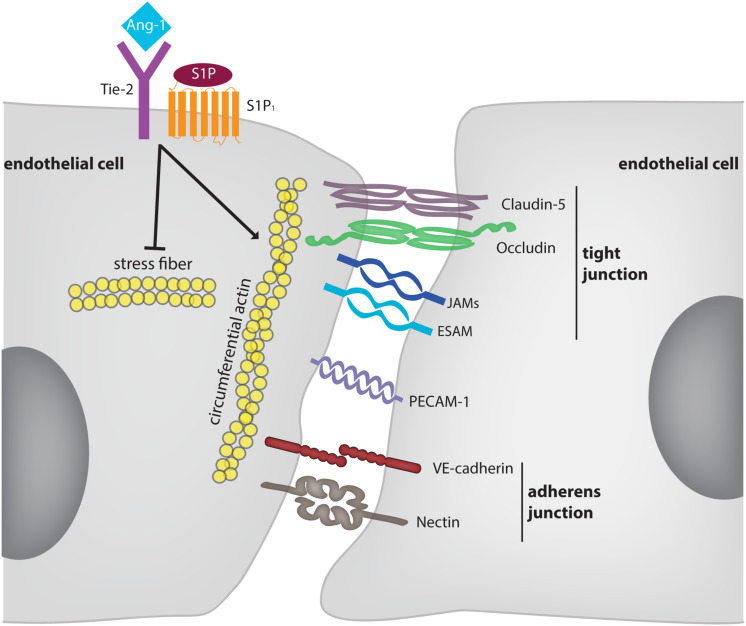
Molecular mechanisms supporting and modulating endothelial junction integrity. The diagram depicts adhesion molecules which contribute to the barrier function of endothelium for solutes. For some of these molecules (e.g., nectins) evidence was only provided *in vitro*. Membrane proteins, such as CD99 and CD99L2 were omitted since they are selectively involved in leukocyte extravasation, but not in junction stability. The receptors Tie-2 and S1P1 are exemplary for mechanisms which stabilize endothelial junctions in an indirect way. These mechanisms are partly dependent and partly independent on VE-cadherin.

The purpose of this review is to highlight, which of the many adhesion molecules at endothelial cell contacts are indeed essential for the formation, the stability and the regulation of endothelial junctions. An overview about the adhesion molecules is given in Figure 1 and Table 1. In addition, we want to discuss organ specific differences. The role of endothelial adhesion molecules in leukocyte transmigration through endothelial barriers will only be marginally addressed and we refer the reader for this topic to recent reviews (Nourshargh and Alon, 2014; Vestweber, 2015; Muller, 2016).

**TABLE 1:** Functions of adhesion molecules at endothelial cell contacts.

Adhesion molecule	Angiogenesis	Baseline junction integrity	Control of vascular permeability induction	Lymph-angiogenesis
PECAM-1	*In vitro* tube formation and *in vivo* matrigel plug assay (Cao et al., 2009)	No defect in endothelial junction integrity in KO mice (Duong et al., 2020)	Enhanced effects of inflammation inducing agents upon blocking or depletion (Ferrero et al., 1995; Graesser et al., 2002; Carrithers et al., 2005; Maas et al., 2005; Liao et al., 2018)	
JAM-A	Matrigel plug assays (Cooke et al., 2006); angiogenesis in corneal wound healing assays (Chatterjee et al., 2013)	No defect in endothelial junction integrity in KO mice (Duong et al., 2020)	Enhanced effects of inflammation inducing agents upon blocking or depletion (Mandell et al., 2006; Mitchell et al., 2015)	
JAM-C	Antibodies and soluble JAM-C interfere with neovascularization in the retina (Lamagna et al., 2005; Hou et al., 2012; Economopoulou et al., 2015)		Reduced effects of inflammation inducing agents upon blocking or depletion (Orlova et al., 2006; Li et al., 2009)	
ESAM	Support of VE-cadherin function in anastomosis in zebrafish embryo (Sauteur et al., 2017); no role in mouse embryonic angiogenesis (Wegmann et al., 2006; Ueda et al., 2019), but tumor angiogenesis and matrigel plug assay (Ishida et al., 2003)	Plasma leaks in lung, but not heart, skin and brain in KO mice (Duong et al., 2020); rupture of endothelial junctions in lung upon simultaneous block of ESAM and VE-cadherin (Duong et al., 2020)	Reduced effects of inflammation inducing agents upon gene inactivation (Wegmann et al., 2006)	
Cldn5		Increased leaks for small molecular weight tracers across BBB, yet no defect in tight junction ultrastructure (Nitta et al., 2003)		
DSG-2	*In vitro* tube formation from endothelial progenitors and *in vivo* matrigel plug assay (Ebert et al., 2016)			
Nectin-2	*In vitro* tube formation (Son et al., 2016)			
VE-cadherin	Embryonic lethality due to defects in vascular remodeling in gene inactivated mice (Carmeliet et al., 1999; Gory-Faure et al., 1999)	Organ specific plasma leaks upon blocking or gene inactivation(Corada et al., 1999; Frye et al., 2015; Duong et al., 2020); rupture of endothelial junctions in lung upon simultaneous block of ESAM and VE-cadherin (Duong et al., 2020)	Relevance of tyrosine phosphorylation for junction regulation (Orsenigo et al., 2012; Wessel et al., 2014)	Organ specific defects upon gene inactivation (Hägerling et al., 2018)

## Composition of Adhesion Molecules at Endothelial Junctions

Endothelial cell contacts are formed and regulated by junctional adhesion molecules that constitute closely associated tight and adherens junctions which mediate and control cell contact integrity and molecular permeability across the endothelial barrier. Adherens junctions are generally considered to provide stability of interendothelial cell contacts and control permeability for large molecular weight plasma components. VE-cadherin represents its major constituent, as antibodies against VE-cadherin or gene inactivation are sufficient to perturb endothelial monolayers *in vitro* and enhance vascular permeability for plasma proteins *in vivo* (Gotsch et al., 1997; Matsuyoshi et al., 1997; Gulino et al., 1998; Corada et al., 1999; Frye et al., 2015; Duong et al., 2020). VE-cadherin is essential for the development of the vascular system (Carmeliet et al., 1999; Gory-Faure et al., 1999) and is probably the adhesion molecule with highest endothelial specificity, with some expression on trophoblasts and fetal stem cells (Aird, 2007a), perineural cells (Colom et al., 2012) and the perineurium of peripheral nerves (Smith et al., 1998; Ouyang et al., 2019).

Nectins are much better studied in epithelial cells than in endothelial cells (Rikitake et al., 2015). Of the classical members, nectin-2 and nectin-3 support endothelial junction integrity *in vitro* (Martin et al., 2013; Son et al., 2016; Bekes et al., 2019), and the nectin related poliovirus receptor (PVR/CD155) is involved in leukocyte extravasation (Reymond et al., 2004).

In contrast to adherens junctions, tight junctions control permeability for ions and small molecules (<800 dalton) and therefore are not evenly distributed throughout the vasculature. They are most prominent in the blood-brain barrier (BBB) and the blood-retina barrier as well as in arterioles (Simionescu et al., 1976; Tietz and Engelhardt, 2015). Central components of tight junctions are claudins (Cldn), tetraspanning membrane proteins, of which Cldn5 is found in vessels in all organs (Morita et al., 1999). Expression of Cldn3, Cldn12, and Cldn1 in brain capillary endothelial cells is rather controversial (Ohtsuki et al., 2008; Tietz and Engelhardt, 2015; Vanlandewijck et al., 2018), and while Cldn3 is inducible during mouse brain angiogenesis (Liebner et al., 2008) it is clearly absent in adult brain endothelium (Castro Dias et al., 2019). Occludin is another bona fide tight junction component of endothelial and epithelial cells, however, its gene inactivation does not cause obvious defects in the vasculature (Saitou et al., 2000). The junctional adhesion molecules (JAMs) are 2 Immunoglobulin-domain proteins of which four are expressed on endothelial cells: JAM-A, JAM-B, and JAM-C and the related endothelial cell-selective adhesion molecule (ESAM). The JAMs are not specific for endothelial cells, with JAM-A and JAM-C being expressed on epithelia and leukocytes and JAM-B on Sertoli cells (Bradfield et al., 2007a). The JAMs are well studied for their role in epithelial barriers and leukocyte extravasation (Bradfield et al., 2007a). ESAM was originally identified as an adhesion molecule specifically expressed on endothelial cells and platelets (Hirata et al., 2001; Nasdala et al., 2002), which is generally not expressed on epithelia, with the exception of the mesothelium (Duong et al., 2020). In addition, ESAM is a marker for primitive hematopoietic progenitors (Ooi et al., 2009; Yokota et al., 2009).

Adhesion molecules at endothelial cell contacts, which are not assigned to junctional complexes are the platelet endothelial cell adhesion molecule (PECAM)-1, CD99 and CD99L2. PECAM-1 is found on endothelial cells, various leukocytes and platelets (Lertkiatmongkol et al., 2016) and on trophoblast cells during invasion of spiral arteries (Blankenship and Enders, 1997). It is very well established for its role in leukocyte diapedesis through the endothelial barrier (Muller, 2011) and plays a role in endothelial cell integrity (Privratsky et al., 2011) as well as in pathological angiogenesis (Cao et al., 2009), although gene inactivated mice do not show vascular defects (Duncan et al., 1999). CD99 and CD99L2 are not specific for the vascular system, they participate in leukocyte extravasation (Vestweber, 2015; Muller, 2016; Li et al., 2019), but are not relevant for endothelial junction integrity or angiogenesis.

## Junctional Adhesion Mechanisms Essential for the Development of the Blood Vascular System

Of the many adhesion molecules at endothelial cell contacts, VE-cadherin is the only one, which is essential for the formation of the vasculature and therefore essential for embryonic development. Gene inactivation of VE-cadherin leads in mice to embryonic lethality at E9.5 due to severe vascular defects (Carmeliet et al., 1999; Gory-Faure et al., 1999). Whereas assembly of endothelial cells in vascular structures is still possible, the subsequent remodeling and maturation is defective, resulting in gross dilation of some vessels and disconnected endothelial cells. Defects were likely related to enhanced apoptosis and lack of survival of endothelial cells (Carmeliet et al., 1999).

For ESAM, a subtle role during segmental artery formation in zebrafish embryos was suggested affecting anastomosis of intersegmental vessels when VE-cadherin was absent (Sauteur et al., 2017). However, no vascular defects were found in single ESAM zebrafish mutants, and no defects in angiogenesis were found in ESAM^–/–^ mouse embryos (Ishida et al., 2003; Wegmann et al., 2006). Pathological angiogenesis in adult ESAM^–/–^ mice such as tumor angiogenesis or neovascularization of implanted matrigel plugs was impaired (Ishida et al., 2003). While ESAM deficiency in mice causes death of half of all ESAM^–/–^ fetuses, this is not due to defects of the vascular system, but caused by impaired definitive hematopoiesis in the fetal liver (Ueda et al., 2019), in line with ESAM being a marker of primitive hematopoietic progenitors (Ooi et al., 2009; Yokota et al., 2009).

PECAM-1 is not essential for embryonic development, yet contributes to angiogenesis in Matrigel plug assays and *in vitro* tube formation assays (Cao et al., 2009). Likewise, occludin and Desmoglein (DSG)-2 are expressed on a subset of endothelial progenitor cells (EPCs) in human cord blood, where they support the formation of tube structures *in vitro* (Ebert et al., 2016; Kanayasu-Toyoda et al., 2018). Also, Nectin-2 supports endothelial tube formation *in vitro* (Son et al., 2016).

## Maintenance of Physical Integrity of Endothelial Junctions in Blood Vessels of the Adult Organism

Analyzing the need of VE-cadherin for maintenance of vascular integrity in the adult organism, it was found that induced gene inactivation of VE-cadherin (Cdh-5^iECKO^) caused vascular leaks for plasma proteins (Frye et al., 2015). However, this effect was limited to only some organs such as heart and lung, and was not observed for skin or brain. Similar organ specific effects were caused by antibodies against VE-cadherin (Corada et al., 1999; Duong et al., 2020). Although subcutaneous bleeding followed by death was caused within several days after intraperitoneally injecting anti VE-cadherin hybridoma cells (Matsuyoshi et al., 1997), similar effects were not seen in Cdh-5^iECKO^ mice, which survived for more than 20 days without VE-cadherin (Frye et al., 2015). Interestingly, neither adhesion blocking antibodies nor gene inactivation caused plasma leaks in dermis or brain (Corada et al., 1999; Frye et al., 2015; Duong et al., 2020). More surprisingly, a detailed analysis by electron microscopy revealed that neither blocking nor gene inactivation of VE-cadherin caused physical rupture of adherens junctions, even in organs where plasma leaks were detected, like in lung and heart (Frye et al., 2015; Duong et al., 2020). This demonstrated, that once endothelial junctions have completely formed in blood vessels of various adult organs, VE-cadherin is not essential anymore for maintenance of their physical integrity. While N-cadherin can replace VE-cadherin in its absence in junctions between cultured endothelial cells (Giampietro et al., 2012; Frye et al., 2015), a similar compensation of the loss of VE-cadherin by N-cadherin could be excluded *in vivo* in VE-cadherin deficient mice (Frye et al., 2015). In addition, other cadherins were excluded since β-catenin was not detectable at endothelial junctions of micro vessels devoid of VE-cadherin (Frye et al., 2015). This is quite remarkable, since it means that, once established, endothelial adherens junctions are stable *in vivo* in the absence of a classical cadherin. This also highlights a central difference between endothelial junctions *in vitro* and *in vivo*. In summary, these studies reveal that, first, only a subset of organs such as lung and heart depend on VE-cadherin to avoid plasma leaks and, second, endothelial junctions do not lose their physical integrity in the absence of VE-cadherin even in organs where plasma leaks occur (Frye et al., 2015). Third, this surprising stability is not due to a replacement of VE-cadherin by other classical cadherins (Frye et al., 2015).

Obviously, other adhesion mechanisms must exist, which support the stability of endothelial junctions. Comparing ESAM, JAM-A and PECAM-1, it was recently shown that gene inactivation of ESAM induced plasma leaks in the lung, but not in heart, skin or brain, whereas no such effects were seen in any of these organs in the absence of JAM-A or PECAM-1 (Duong et al., 2020). Importantly, blocking of VE-cadherin with antibodies in ESAM^–/–^ mice led to almost immediate lethality due to physical rupture of endothelial junctions of the lung vasculature which caused massive thrombosis, whereas no such effects were found in WT mice or mice deficient for JAM-A or PECAM-1 (Duong et al., 2020). Similar effects were seen in this study for VE-cadherin/ESAM double gene deficient mice. Thus, ESAM is essential to prevent plasma leaks in the lung vasculature and is able to prevent physical rupture of endothelial junctions in the lung if VE-cadherin is absent.

## Adhesion Molecules Which Regulate the Barrier Function of Blood Endothelium

In addition to the important role of VE-cadherin for endothelial junction stability, VE-cadherin is also an important and essential target for the regulation of endothelial junction opening and closure. Tyrosine phosphorylation is a key event for the regulation of VE-cadherin function and presence at junctions (Vestweber et al., 2014). Regulation of the phosphorylation of Y685 and Y658 of VE-cadherin were reported to control bradykinin induced endothelial permeability (Orsenigo et al., 2012). Based on the analysis of knock in mice expressing point mutated forms of VE-cadherin instead of WT VE-cadherin it was shown that the induction of vascular permeability by histamine or VEGF depended on the increase of phosphorylation of Y685 of VE-cadherin (Wessel et al., 2014). Interestingly, this tyrosine was not relevant for leukocyte extravasation. Instead, the dephosphorylation of Y731 of VE-cadherin was required for the diapedesis of leukocytes through endothelium *in vivo* (Wessel et al., 2014). Again, this tyrosine was only relevant for leukocyte extravasation but not for the induction of vascular permeability. Thus VE-cadherin is an important target for the opening of endothelial junctions *in vivo*, yet is addressed in different ways for the regulation of permeability and leukocyte diapedesis.

Endothelial cell-selective adhesion molecule also modulates the induction of vascular permeability and leukocyte extravasation *in vivo*, however, in a different way than expected. In ESAM gene deficient mice the induction of vascular permeability by histamine and VEGF in the skin was reduced and the extravasation of leukocytes in cremaster and peritoneum was delayed (Wegmann et al., 2006). Thus, despite its supportive role of junction stability (at least in the microvasculature of the lung), ESAM assists in the opening of junctions under inflammatory conditions. This activity might be related to the support of RhoA activation in endothelial cells (Wegmann et al., 2006). Interestingly, JAM-C was also found to rather support junction destabilization, since interference with JAM-C reduced permeability induction by inflammatory mediators (Orlova et al., 2006). Mechanistically, this study showed that the loss of JAM-C enhanced Rap1 activity. A supportive role for JAM-C for permeability induction was also reported by others, in combination with effects of JAM-C on α_V_β_3_ integrin localization and the activity of Rap1b, but not Rap1a (Li et al., 2009). With respect to leukocyte diapedesis through the endothelium, JAM-C also plays a special role. Blocking its activity with antibodies enhanced reverse transmigration of leukocytes through endothelial junctions (Bradfield et al., 2007b; Woodfin et al., 2011). Furthermore, it was found recently that local microvascular leakage promotes movement of interstitial chemokines into the bloodstream (against the direction of leakage), which supports abluminal-to-luminal transmigration of neutrophils (Owen-Woods et al., 2020).

Despite the lack of an essential role of PECAM-1 or JAM-A for endothelial junction stability in the absence of pathological challenge, a regulatory role under inflammatory settings has been established for PECAM-1. It supports maintenance of the endothelial barrier against inflammatory challenges *in vivo* (Ferrero et al., 1995; Graesser et al., 2002; Carrithers et al., 2005; Maas et al., 2005). In addition, the cytoplasmic domain of PECAM-1 supports the barrier integrity of cultured endothelial cells and recovery of junctions upon dissociation with thrombin (Liao et al., 2018). The role of JAM-A for the formation and stability of epithelial cells is very well established (Laukoetter et al., 2007), whereas its role for endothelial junctions is less well studied. In rabbits it was shown that antibodies against JAM-A caused corneal swelling and impaired junction reformation in cultured corneal endothelial cells in calcium depletion assays (Mandell et al., 2006) and LPS-induced pulmonary edema was enhanced in JAM-A^–/–^ mice (Mitchell et al., 2015).

JAM-A, PECAM-1, CD99, and CD99L2 and the Nectin PVR (CD155) are all well established as supporters of leukocyte extravasation, which is discussed elsewhere (Nourshargh and Alon, 2014; Vestweber, 2015; Muller, 2016; Maas et al., 2018).

Claudins determine the selective permeability of tight junctions for ions or small molecular weight tracers. New born Cldn5-deficient mice showed increased passage of such tracers across the blood brain barrier, whereas the ultrastructure of tight junctions were unaffected (Nitta et al., 2003) and in a model of ischemic stroke, Cldn5 was targeted by matrix metalloproteinases (Yang et al., 2007).

Besides adhesion molecules, also the tyrosine kinase receptors Tie-2 and the sphingosine 1-phosphate receptor 1 (S1P_1_) stabilize endothelial junctions and counteract inflammation induced vascular permeability (Thurston et al., 1999; Wang and Dudek, 2009). For both receptor systems it was shown *in vitro* that stimulation enhanced the presence of VE-cadherin at endothelial junctions or counteracted its endocytosis (Lee et al., 1999; Gavard et al., 2008). In addition, S1P had barrier enhancing effects *in vitro* even when VE-cadherin was blocked or removed (Xu et al., 2007) and endotoxin induced vascular permeability in the mouse lung could be counteracted by Tie-2 activation even in mice gene inactivated for VE-cadherin (Frye et al., 2015). Furthermore, interference with Tie-2 expression *in vivo* enhanced baseline vascular permeability in the lung, arguing that Tie-2 is also contributing to baseline vascular integrity (Frye et al., 2015). Tie-2 and S1P_1_ stabilize endothelial junctions by modulating actomyosin fiber tension of radial stress fibers and circumferential actin via the regulation of Rac1, Cdc42, and Rho (Mammoto et al., 2007; Wang and Dudek, 2009; David et al., 2011; Frye et al., 2015; Braun et al., 2019). In addition, actin polymerization and dynamics regulate junction formation and stability (Cao and Schnittler, 2019).

Integrins can also indirectly affect endothelial junction integrity by mechanisms which still need more investigation. Gene inactivation of the β_1_-integrin chain impaired proper localization of VE-cadherin and thereby endothelial junction integrity *in vivo* (Yamamoto et al., 2015). In agreement with this, talin dependent integrin activation was reported to regulate VE-cadherin localization and endothelial barrier function (Pulous et al., 2019). The Abl kinase inhibitors imatinib and bosutinib were found to prevent LPS-induced alveolar protein extravasation in the lung and acted on junctions potentially via reducing the turnover of integrin supported focal adhesions (Aman et al., 2012; Botros et al., 2020).

## Development and Maintenance of Lymphatic Endothelial Junctions

The lymphatic vasculature takes up extravasated fluid and cells and transports them back to the blood circulation (Alitalo et al., 2005; Tammela and Alitalo, 2010; Koh and Petrova, 2018). Fluid entry occurs through blunt-ending initial lymphatics. Their junctions differ from those of lymphatic collectors by overlapping flaps at cell contacts, which allow fluid entry and are anchored on their sides by button like junctions (Baluk et al., 2007). While the button-like junctions contain VE-cadherin, ESAM, JAM-A, Cldn5 and occludin, the flap-like areas contain PECAM-1 (Baluk et al., 2007). Button junctions appear just before birth (Yao et al., 2012). The coxsackie- and adenovirus receptor (CAR), a protein related to the JAMs and ESAM, was described at junctions of human dermal lymph endothelial cells (Vigl et al., 2009) and its induced deletion at E12.5, but not at a later time point in development, caused dilation of subcutaneous lymphatic vessels and edema (Mirza et al., 2012).

VE-cadherin is indispensable for the development of certain lymph vessel beds (Hägerling et al., 2018). During embryonic development, deletion of VE-cadherin in lymphatic endothelial cells caused impaired lymphangiogenesis and embryonic lethality. Induced gene deletion postnatally or in the adult organism caused different effects in different organs. VE-cadherin deletion in adult mice did not impair dermal lymphatics and left button-like junctions intact. Although some adhesion molecules such as PECAM-1, JAM-A, and JAM-C were upregulated, N-cadherin expression was not affected and was not found in button like junctions. In contrast to dermal lymphatics, mesenteric lymph vessels were much more sensitive to the loss of VE-cadherin, which caused mesenteric lymph vessel disintegration, even in adults (Hägerling et al., 2018). This was accompanied by hyperproliferation of lymphatic endothelial cells in mesenteries. In addition, lymphatic valve formation was generally affected by deletion of VE-cadherin (Hägerling et al., 2018; Yang et al., 2019).

Button like lymph endothelial junctions are essential for fluid uptake. The formation of these special junctions requires the Tie-2 ligand Angiopoietin-2, and was accompanied by the phosphorylation of Y685 of VE-cadherin (Zheng et al., 2014). The induced deletion of neuropilin 1 and FLT1 was recently described as a genetic defect which prevents the formation of button like junctions by enhancing the availability of VEGF-A (Zhang et al., 2018). As a physiological inducer, the gut microbiota was discovered as an essential stimulator of VEGF-C which is needed for the formation of button like junctions in initials of the intestine (lacteals) (Suh et al., 2019).

## Conclusion

As outlined above, VE-cadherin is certainly a major player for the formation and maintenance of endothelial junctions, in the blood as well as the lymphatic vasculature. Yet, fully established endothelial junctions can be maintained *in vivo* in the absence of VE-cadherin and even any other classical cadherin at least for weeks without physical rupture. Recent evidence established that it is ESAM which prevents rupture of vascular junctions in the lungs of these mice. Since ESAM is a tight junction-associated molecule, this raises the question how tight and adherens junctions cooperate with each other in providing vascular junction stability. In addition, we need a better understanding of the processes that regulate radial acto-myosine stress fibers and circumferential acto-myosine in cooperation with the anchoring of junctional adhesion molecules and their outside in signaling. Furthermore, crosstalk between integrin dependent focal adhesion turn over and junction stability is a newly emerging topic that is important for the understanding of endothelial junction dynamics and regulation of vascular permeability. Finally, the relevance of certain adhesion molecules and signaling mechanisms for the integrity of endothelial junctions clearly varies between different tissues, which will be an important aspect to study in the future.

## Author Contributions

DV and CD wrote and edited the manuscript.

## Conflict of Interest

The authors declare that the research was conducted in the absence of any commercial or financial relationships that could be construed as a potential conflict of interest.
